# Food-associated cues alter forebrain functional connectivity as assessed with immediate early gene and proenkephalin expression

**DOI:** 10.1186/1741-7007-5-16

**Published:** 2007-04-26

**Authors:** Craig A Schiltz, Quentin Z Bremer, Charles F Landry, Ann E Kelley

**Affiliations:** 1 Medical Scientist and Neuroscience Training Programs, University of Wisconsin-Madison School of Medicine and Public Health, 6001 Research Park Boulevard, Madison, WI 53705, USA; 2 Department of Psychiatry, University of Wisconsin-Madison School of Medicine and Public Health, 6001 Research Park Boulevard, Madison, WI 53719, USA

## Abstract

**Background:**

Cues predictive of food availability are powerful modulators of appetite as well as food-seeking and ingestive behaviors. The neurobiological underpinnings of these conditioned responses are not well understood. Monitoring regional immediate early gene expression is a method used to assess alterations in neuronal metabolism resulting from upstream intracellular and extracellular signaling. Furthermore, assessing the expression of multiple immediate early genes offers a window onto the possible sequelae of exposure to food cues, since the function of each gene differs. We used immediate early gene and proenkephalin expression as a means of assessing food cue-elicited regional activation and alterations in functional connectivity within the forebrain.

**Results:**

Contextual cues associated with palatable food elicited conditioned motor activation and corticosterone release in rats. This motivational state was associated with increased transcription of the activity-regulated genes *homer1a*, *arc*, *zif268*, *ngfi-b *and c-*fos *in corticolimbic, thalamic and hypothalamic areas and of proenkephalin within striatal regions. Furthermore, the functional connectivity elicited by food cues, as assessed by an inter-regional multigene-expression correlation method, differed substantially from that elicited by neutral cues. Specifically, food cues increased cortical engagement of the striatum, and within the nucleus accumbens, shifted correlations away from the shell towards the core. Exposure to the food-associated context also induced correlated gene expression between corticostriatal networks and the basolateral amygdala, an area critical for learning and responding to the incentive value of sensory stimuli. This increased corticostriatal-amygdalar functional connectivity was absent in the control group exposed to innocuous cues.

**Conclusion:**

The results implicate correlated activity between the cortex and the striatum, especially the nucleus accumbens core and the basolateral amygdala, in the generation of a conditioned motivated state that may promote excessive food intake. The upregulation of a number of genes in unique patterns within corticostriatal, thalamic, and hypothalamic networks suggests that food cues are capable of powerfully altering neuronal processing in areas mediating the integration of emotion, cognition, arousal, and the regulation of energy balance. As many of these genes play a role in plasticity, their upregulation within these circuits may also indicate the neuroanatomic and transcriptional correlates of extinction learning.

## Background

Daily consumption of a mere 50–100 extra kilocalories without compensatory expenditure has been proposed to be a main cause of the increasing incidence of overweight and obesity [1,2]. As this trend has been occurring over the past 25 years, environmental factors such as increased food availability and the power of sensory food-associated sensory cues in the modern environment (e.g. advertising) may ultimately be responsible for the initiation and maintenance of increased energy intake [3].

It has been known for some time that food-related cues are capable of increasing food consumption beyond homeostatic needs and that this effect is enhanced when subjects are hungry [4-9]. Functional imaging studies indicate that regional cortical, limbic, striatal, thalamic or hypothalamic activation in response to presentation of food images is greater in food-restricted than in satiated subjects [10-12]. Subjects will also increase their intake of a preferred food, but not other foods, when they are presented with sensory cues specific to that food [7,13]. Furthermore, presentation of cues associated with preferred versus non-preferred foods increases activity in cortical areas and the amygdala [10,14,15], and images of high-calorie versus low-calorie foods activate regions of the cortex and the thalamus [16,17]. Overall, these studies demonstrate that food preference, caloric density, and short-term energy balance can influence regional neuronal activity and food intake resulting from exposure to food-related stimuli. Interestingly, stress can also increase self-reported liking and overall intake of food, yet the neuroanatomical substrates of these phenomena remain largely uninvestigated [18-21].

**Figure 1:**
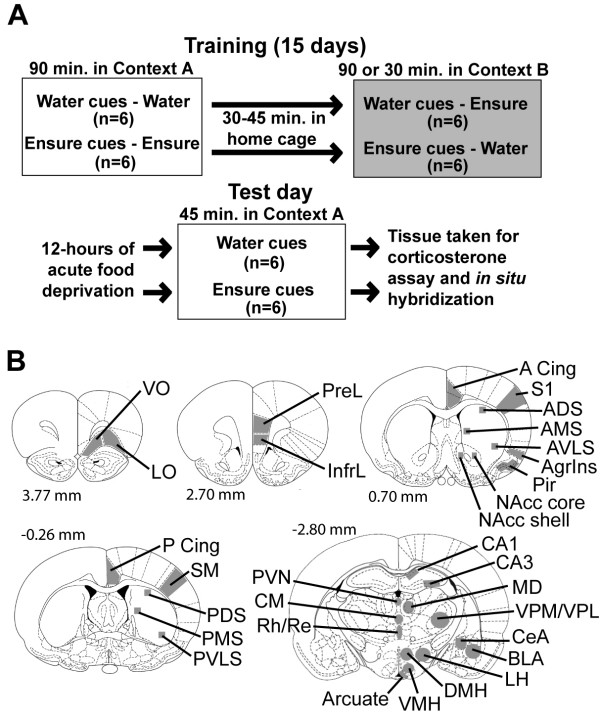
**Schematic diagrams of the experimental paradigm and the brain regions analyzed for gene expression**. (**A**) The experimental paradigm consisted of a 15-day training period in which all rats received access to chocolate Ensure and water in one of two distinct environments (contexts A and B). Locomotor activity was measured in context A over the training phase of the study. On the test day, 3 days after the final day of training, rats were reintroduced to context A after an acute 12-hour food deprivation period. Locomotor activity was measured in context A for 45 minutes, after which time rats were killed, and trunk blood and brains were taken for plasma corticosterone assay and *in situ *hybridization analysis of forebrain gene expression. (**B**) The brain regions analyzed for activity-dependent gene expression via *in situ *hybridization included the ventral orbital prefrontal cortex (VO), lateral orbital prefrontal cortex (LO), prelimbic cortex (PreL), infralimbic cortex (InfrL), anterior and posterior cingulate cortex (A and P Cing), primary sensory cortex (S1), agranular insular cortex (AgrIns), piriform cortex (Pir), sensorimotor cortex, nucleus accumbens shell (NAcc shell), nucleus accumbens core (NAcc core), anterior ventrolateral striatum (AVLS), anterior medial striatum (AMS), anterior dorsolateral striatum (ADLS), sensorimotor cortex (SM), posterior ventrolateral striatum (PVLS), posterior medial striatum (PMS), posterior dorsolateral striatum (PDLS), CA1 and CA3 subfields of the dorsal hippocampus, basolateral amygdala (BLA), central nucleus of the amygdala (CeA), periventricular thalamus (PVN), centromedian thalamus (CM), rhomboid and reunions thalamus (Rh/Re), mediodorsal thalamus (MD), ventralposteromedial and ventralposterolateral thalamus (VPM/VPL), lateral hypothalamus (LH), dorsomedial hypothalamus (DMH), ventromedial hypothalamus (VMH), and arcuate hypothalamus. Numbers represent distance from bregma in mm (adapted with permission from [131]).

While it is clear that increased food intake results from exposure to food-related stimuli and stressors, little is known about the functional circuits mediating this behavior. To further characterize the ability of food-associated cues to activate central circuits, we employed a classical conditioning paradigm in which rats were exposed to an environment that had previously been repeatedly paired with access to chocolate Ensure (a palatable, energy-dense liquid; Abbott Laboratories, OH, USA) or to control cues (Figure 1A). *In situ *hybridization (ISH) for the immediate early genes (IEGs) *homer1a*, *arc*, *zif268*, *ngfi-b*, and *c-fos *were used to map alterations in neuronal activity in cortical, striatal, thalamic, and hypothalamic regions (Figure 1B). The early response genes fall into different subclasses serving differing functions, therefore parallel or divergent changes in their expression offer insight into specific cellular and molecular processes occurring within individual brain regions. For example, the Arc and Homer1a proteins are involved in trafficking α-amino-3-hydroxy-5-methyl-4-isoxazole propionic acid (AMPA) and metabotropic glutamate receptors, respectively [22-29]. Zif268, NGFI-B, and c-Fos are transcription factors with differing promoter specificities. Measuring the expression of multiple IEGs also allowed us to perform a statistically powerful interregional gene expression correlation analysis to identify functional circuits recruited by food-associated cues compared with control-associated cues.

Our findings demonstrate that cues associated with palatable food elicited a state of motivational arousal associated with robust IEG expression within the prosencephalon, including basal diencephalic areas responsible for the detection of peripheral energy homeostasis signals and integration of these signals into autonomic and behavioral outputs. The differing patterns of correlated gene expression elicited by food-associated versus control-associated cues suggests that the generation of this state involved major shifts in functional connectivity between cortical, amygdalar, and striatal regions. In addition, we found that striatal expression of proenkephalin, an endogenous opioid that plays a role in food consumption and cue-elicited reward-seeking behavior, was enhanced by food-associated cues [30-39].

## Results

**Figure 2:**
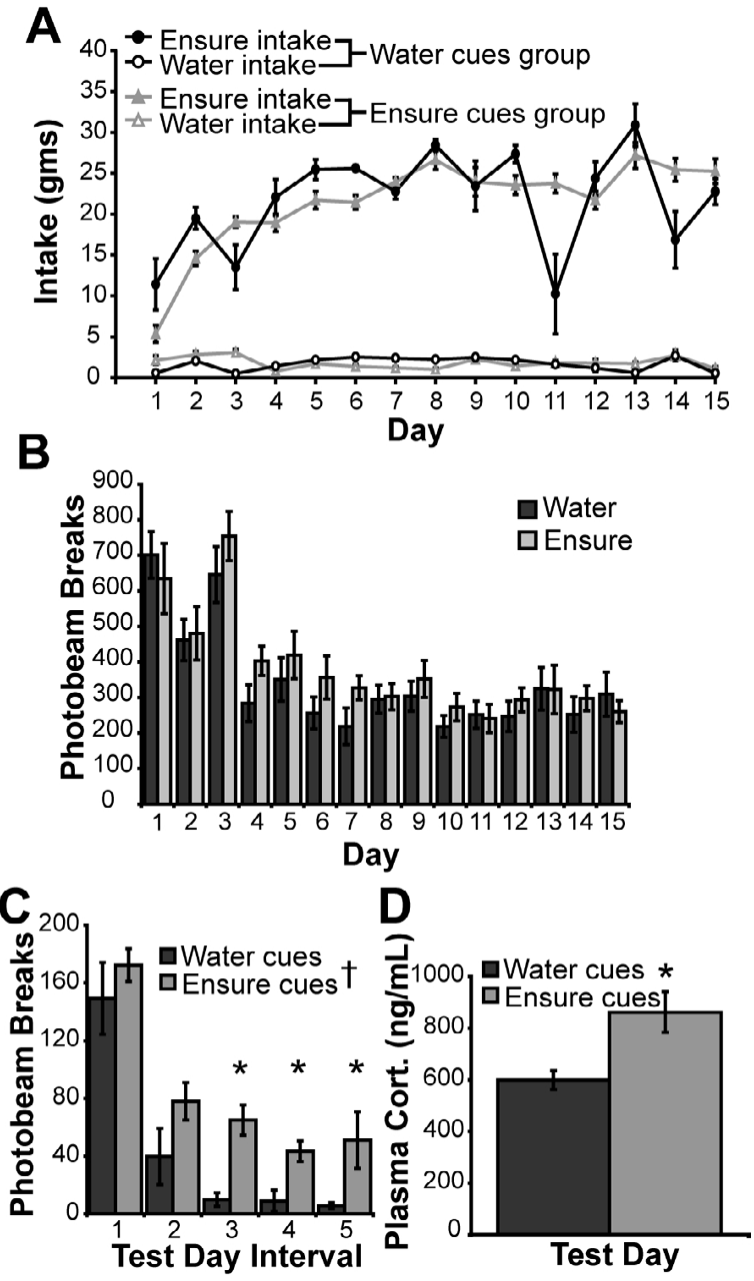
**Behavioral and endocrine data**. (**A**) Intake of Ensure and water by the water cues and Ensure cues groups in their respective contexts. Ensure intake was significantly greater than water intake and was not different between the groups (**p < 0.01). Ensure intake increased and reached a plateau over the 15 day training period (††*p *< 0.01). (**B**) Locomotor activity in response to access to water or Ensure in context A during daily training sessions. The levels of total horizontal activity between the groups did not differ significantly, and decreased during the training period, demonstrating that both groups habituated to context A despite different treatments in this environment. (**C**) While there was no difference in the patterned relationship of total horizontal activity with time between the groups, total horizontal activity was significantly higher on the test day in the Ensure cues group than in the water cues group during intervals 3, 4, and 5 (*p < 0.05). (**D**) Levels of plasma corticosterone in Ensure cues group were higher than in the water cues group (*p < 0.05).

In a counterbalanced design, rats fed *ad libitum *on standard chow received access to Ensure, a palatable, high-calorie liquid, or water in one of two environments with distinct sensory cues (contexts A or B) for 15 days (Figure 1A). Three days after the end of training, rats were acutely food-deprived for an average of 12.75 hours and then placed in context A, but received neither water nor Ensure in order to assess conditioned behavioral, endocrine, and regional cerebral transcriptional responses to food-associated cues (Figure 1B). The daily intake of Ensure and water in contexts A and B is shown in Figure 2A. Rats in both the Ensure cues and water cues groups drank significantly more Ensure than water in their respective contexts over the training period (F_1,20 _= 2555.346, *p *< 0.0001). Rats consumed very little water, as they were not water deprived. There was no observed significant effect of context on either Ensure or water intake between the groups (F_1,20 _= 0.0005, *p *= 0.9825) and there was no observed significant interaction between Ensure or water intake and context (F_1,20 _= 0.113, *p *= 0.743). The Ensure and water cues groups initially consumed increasing daily amounts of Ensure, as illustrated by a significant effect of training day on intake (F_14,280 _= 14.825, *p *< 0.0001) and an interaction between training day and intake (F_14,280 _= 15.334, *p *< 0.0001; Figure 2A). Rats in both groups gained weight over the course of the experiment without significant difference between the groups (F_1,10 _= 3.015, *p *= 0.1132).

During the 15-day training period, access to Ensure had no significant stimulatory effect on total horizontal activity in context A compared with locomotor activity in the group receiving water access in this context (Figure 2B). There was a significant effect of day on total horizontal activity (F_14,140 _= 34.117, *p *< 0.0001), indicating a gradual habituation to the environment but there was no treatment × day interaction, as activity in both groups declined similarly over days.

On the test day, rats in the Ensure cues group exhibited higher levels of total horizontal activity compared with rats in the water cues group (F_1,10 _= 9.010, *p *= 0.0133; Figure 2C). There was a significant decrease in locomotor activity over the intervals (F_1,4 _= 47.652, *p *< 0.0001) but no interaction between treatment and interval (F_4,40 _= 0.536, *p *= 0.7102). Total horizontal activity was significantly different between the groups during intervals 3 (F_1,10 _= 23.174, *p *= 0.0007), 4 (F_1,10 _= 11.077, *p *= 0.0076) and 5 (F_1,10 _= 5.323, *p *= 0.0437) but not during intervals 1 (F_1,10 _= 0.707, *p *= 0.4202) or 2 (F_1,10 _= 2.640, *p *= 0.1353). There was also a conditioned increase in plasma corticosterone in the Ensure cues group on the test day (F_1,9 _= 7.953, *p *= 0.0200; Figure 2D).

### Food cues induced immediate early gene expression in the telencephalon

**Figure 3:**
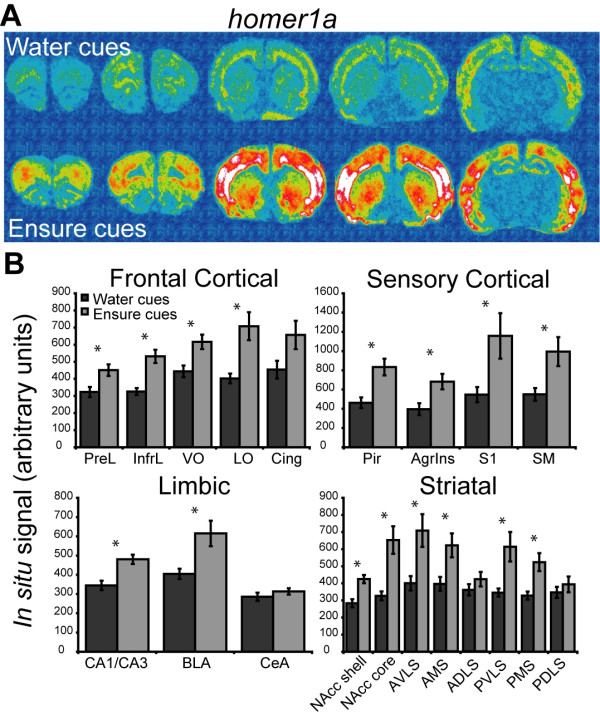
**Telencephalic expression of *homer1a *induced by exposure to water or Ensure cues**. (**A**) Pseudocolor autoradiographic phosphorescence images of coronal brain sections hybridized with a probe for *homer1a *from a rat in the water cues group (top) and the Ensure cues group (bottom). (**B**) Graphical representation of semiquantitative measurements of *in situ *hybridization for *homer1a *in telencephalic regions. Ensure cues increased the expression of *homer1a *in a many of the corticolimbic regions examined (*p < 0.05). For abbreviations, see legend to Figure 1.

Ensure cues elicited *homer1a *expression in a variety of forebrain regions (Figure 3). The overall between-subjects and within-subjects analysis of the *homer1a *data revealed a significant effect of context (F_1,10 _13.218, *p *= 0.0046) and a significant context × region interaction (F_19,190 _3.670, *p *< 0.0001). Further analysis revealed significant increases in *homer1a *in all of the cortical regions analyzed except for the cingulate cortex (see Additional file 1 for a table of the results of one-way ANOVA analyses for all genes). An increase in *homer1a *expression was also found in limbic regions including the dorsal hippocampus and the basolateral amygdala, but not in the central nucleus of the amygdala. Significant increases in *homer1a *expression were observed in striatal regions including the nucleus accumbens core and shell and the ventral and medial regions of the striatum.

The expression pattern of *arc *was similar to that of *homer1a*. Ensure cues increased *arc *levels in a variety of forebrain regions (see Additional file 2). The overall between-subjects and within-subjects analysis of the *arc *data revealed a significant context effect (F_1,10 _29.538, *p *= 0.0003) and a significant context × region interaction (F_19,190 _11.237, *p *< 0.0001). There were significant increases in *arc *in all of the cortical regions analyzed. Ensure cues increased *arc *expression in limbic regions including the dorsal hippocampus and the basolateral amygdala, but not in the central nucleus of the amygdala. In addition, *Arc *was also induced regionally in the striatum.

**Figure 4:**
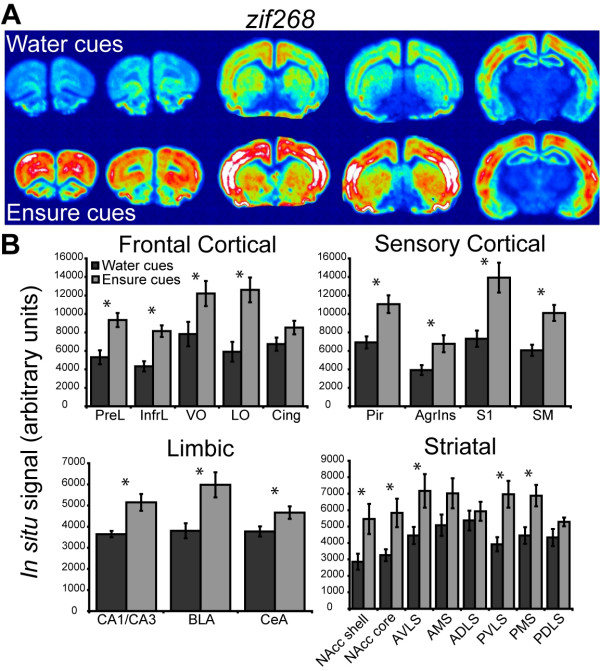
**Telencephalic expression of *zif268 *induced by exposure to water or Ensure cues**. (**A**) Pseudocolor autoradiographic phosphorescence images of coronal brain sections hybridized with a probe for *zif268 *from a rat in the water cues group (top) and the Ensure cues group (bottom). (**B**) Graphical representation of semiquantitative measurements of *in situ *hybridization for *zif268 *in telencephalic regions. Ensure cues increased the expression of *zif268 *in a many of the corticolimbic regions examined (*p < 0.05). For abbreviations, see legend to Figure 1.

The mRNA levels of *zif268 *were increased in the forebrains of rats in the Ensure cues group (Figure 4). The overall between-subjects and within-subjects analysis of the *zif268 *data revealed a significant effect of context (F_1,10 _17.877, *p *= 0.0017) and a significant context × region interaction (F_28,280 _5.252, *p *< 0.0001). Further analysis revealed significant increases in *zif268 *in the Ensure cues group in all of the cortical regions analyzed except the cingulate cortex. Ensure cues also increased *zif268 *expression in limbic regions including the dorsal hippocampus, the basolateral amygdala, and the central nucleus of the amygdala. Significant increases in *zif268 *expression were observed in regions of the striatum including the shell and core of the nucleus accumbens.

Ensure cues increased *ngfi-b *mRNA levels in telencephalic regions (see Additional file 3). The overall between-subjects and within-subjects analysis of the *ngfi-b *data revealed a significant effect of context (F_1,10 _31.485, *p *= 0.0002) and a significant context × region interaction (F_28,280 _12.060, *p *< 0.0001). Further analysis revealed significant increases in *ngfi-b *in several prefrontal and primary sensory cortical areas. Ensure cues also increased *ngfi-b *expression in limbic regions including the dorsal hippocampus and the basolateral amygdala but not in the central nucleus of the amygdala. Striatal regions also exhibited *ngfi-b *induction in response to Ensure cues.

Food cues increased *c-fos *in telencephalic areas (see Additional file 4). Measurement of *c-fos *expression in frontal cortical regions was limited to the cingultate cortex, because more anterior sections were lost during processing. The overall between-subjects and within-subjects analysis of the *c-fos *data revealed a significant context effect (F_1,10 _51.787, *p *< 0.0001) and a significant context × region interaction (F_24,240 _11.848, *p *< 0.0001). Regional analysis revealed significant increases in *c-fos *in the cingulate cortex and primary sensory cortical areas. Similarly, *c-fos *expression was increased in the limbic regions including the dorsal hippocampus, the basolateral amygdala, and the central nucleus of the amygdala. The striatum exhibited region-specific increased *c-fos *levels in response to Ensure cues.

### Food cues induced immediate early gene expression in the diencephalon

**Figure 5:**
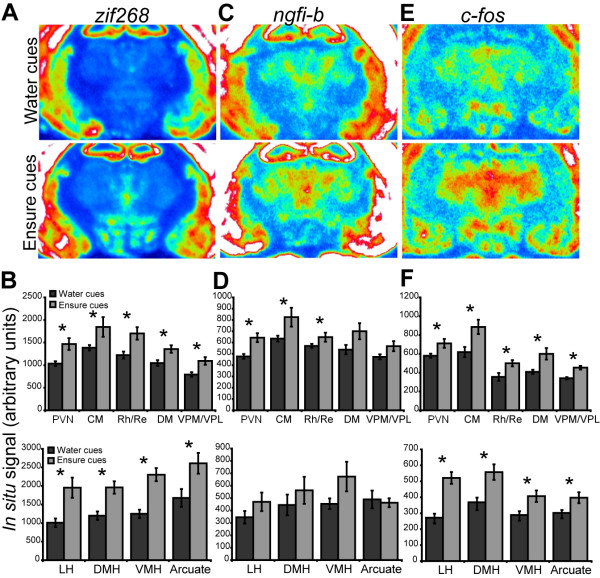
**Diencephalic expression of the transcription factor immediate early genes induced by exposure to water or Ensure cues**. (**A**) Pseudocolor autoradiographic phosphorescence image of a coronal brain sections at the level of the diencephalons, hybridized with a probe for *zif268 *from a rat in the water cues group (top) and the Ensure cues group (bottom). (**B**) Graphical representation of semiquantitative measurements of *in situ *hybridization for *zif268 *in diencephalic regions. Ensure cues increased the expression of *zif268 *in thalamic and hypothalamic regions (*p < 0.05). (**C**) Pseudocolor autoradiographic phosphorescence images of coronal brain sections at the level of the diencephalon hybridized with a probe for *ngfi-b *from a rat in the water cues group (top) and the Ensure cues group (bottom). (**D**) Graphical representation of semiquantitative measurements of *in situ *hybridization for *ngfi-b *in diencephalic regions. Ensure cues increased the expression of *ngfi-b *in thalamic but not hypothalamic regions (*p < 0.05). (**E**) Pseudocolor autoradiographic phosphorescence image of a coronal brain sections at the level of the diencephalon hybridized with a probe for *c-fos *from a rat in the water cues group (top) and the Ensure cues group (bottom). (**F**) Graphical representation of semiquantitative measurements of *in situ *hybridization for *c-fos *in diencephalic regions. Ensure cues increased the expression of *c-fos *in thalamic and hypothalamic regions (*p < 0.05). For abbreviations, see legend to Figure 1.

Only *zif268*, *ngfi-b*, and *c-fos *were detected at appreciable levels within the analyzed diencephalic regions. Expression of z*if268 *was increased in all of the analyzed thalamic and hypothalamic regions in response to Ensure cues (Figure 5A,B), while *ngfi-b *was increased by Ensure cues in some thalamic regions but not in any of the hypothalamic regions analyzed (Figure 5C,D). Ensure cues increased *c-fos *in all of the analyzed thalamic and hypothalamic regions (Figure 5E,F).

### Food cues elicited fewer regional gene expression correlations with locomotion and more correlations with plasma corticosterone

In order to assess whether regional gene-expression measurements have a context-dependent, patterned relationship with locomotor activity, plasma corticosterone, or gene expression within other regions, we performed a series of correlation analyses. The results indicated that food-associated cues changed the relationship of regional activity with conditioned locomotor activity and plasma corticosterone levels and dramatically altered the pattern of functional connectivity.

**Figure 6:**
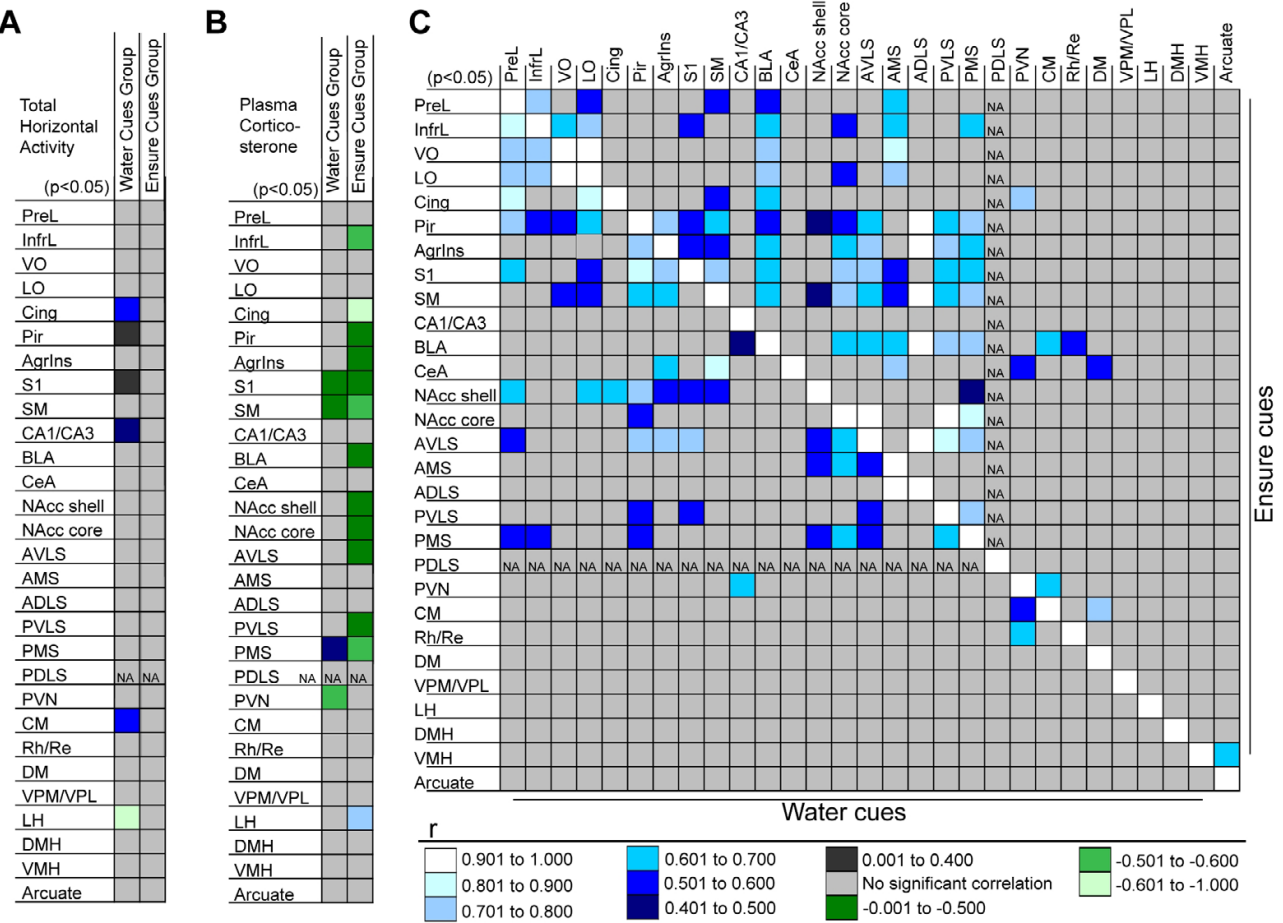
**Graphical representation of the results of correlation analyses in the Ensure and water cues groups**. Collapsed values for the expression of each of the immediate early genes that were normalized to regional control averages were used to calculate correlations within each group so long as there was a significant mean difference between the groups for each individual gene. Correlations that were significant are color-coded by the range in which their Pearson product moment value (r) lies (see key). Correlations that were not significant are indicated by light gray shading. (**A**) Correlations between regional immediate early gene expression and total horizontal activity in the water cues group (left column) and the Ensure cues group (right column). (**B**) Correlations between regional immediate early gene expression and plasma corticosterone in the water cues group (left column) and the Ensure cues group (right column). (**C**) Interregional gene expression correlations in the water cues group (lower left half) and the Ensure cues group (upper right half). NA designates a region for which correlations were not performed due to a lack of significant differences between the groups in all of the genes analyzed. The lack of interregional gene-expression correlations within the diencephalon most likely represents a decrease in the statistical power of the correlation analyses, as only a subset of the genes exhibited significant differences in expression within these regions. For abbreviations, see legend to Figure 1.

Test day locomotor activity was less often correlated with regional early-response gene expression in the Ensure cues group compared with the water cues group (Figure 6A). Total horizontal activity was not significantly correlated with gene expression in any of the analyzed regions in the Ensure cues group. In contrast, within the water cues group, there were several markedly positive correlations between total horizontal activity and gene expression in the cingulate, piriform, and agranular insular cortices and the dorsal hippocampus and centromedial region of the thalamus. There was also a significant negative correlation between total horizontal activity and gene expression in the lateral hypothalamus within this group.

In direct contrast, test day plasma corticosterone levels were more often correlated, albeit inversely, with regional gene expression in the Ensure cues group than in the water cues group (Figure 6B). Early response gene levels in the infralimbic, cingulate, piriform, agranular insular, primary sensory, and sensorimotor cortices, the basolateral amygdala, nucleus accumbens shell and core, anterior and posterior ventrolateral striatum, and the posterior medial striatum all exhibited significant negative correlations with plasma corticosterone in response to Ensure cues. The only region to exhibit a positive correlation between gene expression and plasma corticosterone levels in the Ensure cues group was the lateral hypothalamus.

### Food cues altered interregional gene expression correlations

The pattern of interregional gene expression correlations was markedly different between the Ensure and water cues groups (Figure 6C). The total number of correlations increased, while the number of intracortical and intrastriatal correlations decreased in the Ensure cues group compared with the control cues group. Primary sensory cortical engagement of the striatum almost completely accounted for the increased number of corticostriatal correlations induced by Ensure cues. There was also a shift in the distribution of cortical correlations between the two nucleus accumbens regions. Cortical regions correlated primarily with the nucleus accumbens core in the Ensure cues group and mainly with the nucleus accumbens shell in the water cues group.

Another striking difference between the groups was the pattern of inter-regional gene-expression correlations with the basolateral amygdala (Figure 6C). In the Ensure cues group, gene expression in the basolateral amygdala was correlated with all of the analyzed cortical and striatal areas (except for the nucleus accumbens shell) and was not correlated with the dorsal hippocampus. In contrast, only the dorsal hippocampus and the periventricular region of thalamus exhibited correlations with the basolateral amygdala in the water cues group.

### Food cues increased striatal hnPENK expression

**Figure 7:**
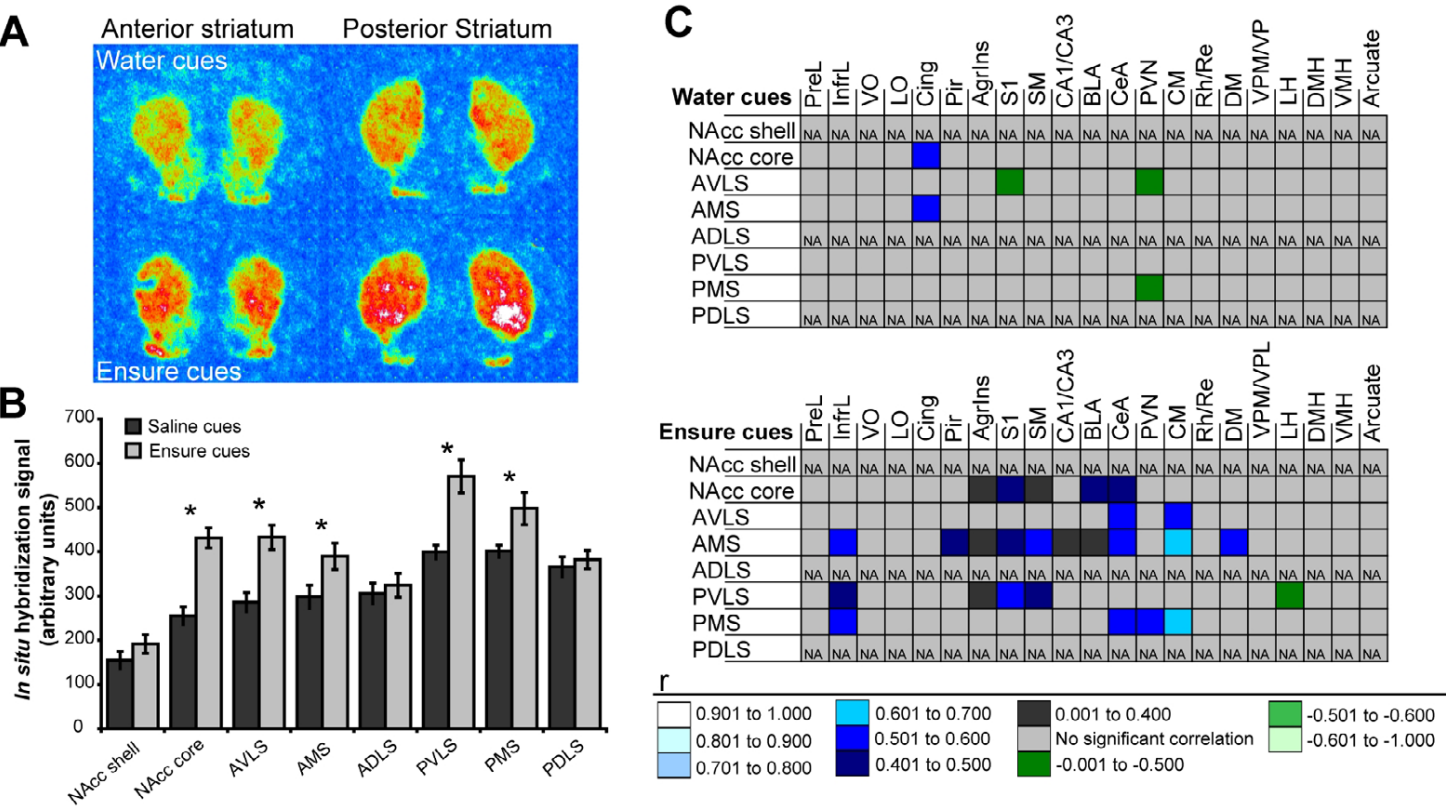
**Striatal expression of *hnPENK***. (**A**) Pseudocolor autoradiographic phosphorescence image of a coronal brain sections at the level of the anterior (left) and posterior (right) striatum hybridized with a probe for *hnPENK *from a rat in the water cues group (top) and the Ensure cues group (bottom). (**B**) Graphical representation of semiquantitative measurements of *in situ *hybridization for *hnPENK *in striatal regions. Ensure cues increased the expression of *hnPENK *in the nucleus accumbens core and ventrolateral and medial regions, but not in the nucleus accumbens shell and dorsolateral regions (*p < 0.05). (**C**) Graphical representation of the results of extrastriatal immediate early gene expression and striatal *hnPENK *expression correlation analyses in both the water cues and the Ensure cues groups. Correlations that were significant are color-coded by the range in which their Pearson product moment (r) value lies (see key). Correlations that are not significant are shaded light gray. For abbreviations, see legend to Figure 1.

Appreciable expression of *hnPENK *was limited to the striatum (Figure 7). The overall between-subjects and within-subjects analysis of the *hnPENK *data revealed a significant context effect (F_1,10_20.861, *p *= 0.0010), and a significant context × region interaction (F_7,70 _4.673, *p *= 0.0002). Significant increases in *hnPENK *expression were restricted to the nucleus accumbens core, anterior and posterior ventrolateral and medial aspects of the striatum. Total horizontal activity, irrespective of group, correlated positively with *hnPENK *expression in the nucleus accumbens core (R = 0.677; *n *= 12; Z = 2.471; *p *= 0.0135) but there were no such correlations in either group analyzed individually.

As cortical or thalamic deafferentation decreases striatal PENK mRNA expression [40,41], we identified potential afferents regulating striatal *hnPENK *in this study by performing correlations between extrastriatal, regional IEG expression and striatal, regional *hnPENK *expression (Figure 7C). Overall, there were 25 positive correlations between extrastriatal immediate early gene expression and striatal *hnPENK *expression in the Ensure cues group versus 2 correlations in the water cues group. These correlations were distributed in a manner consistent with anatomic patterns of cortical and thalamic innervation of the striatum.

## Discussion

We found that contextual cues associated with access to palatable food are capable of eliciting conditioned increases in locomotion and plasma corticosterone. This state is associated with increased IEG expression in cortical, limbic, striatal, thalamic, and hypothalamic areas. In addition, the conditioned stimuli increased striatal transcription of the *PENK *gene, which encodes endogenous opioid neuropeptides. Furthermore, this is the first study correlating the regional expression of multiple, activity-regulated genes as a means of probing functional connectivity in relation to motivation. The differing correlated patterns of gene expression suggest that food cues are capable of powerfully changing functional connectivity within circuits mediating emotion, cognition, arousal, and energy balance, which are used to guide goal-directed behavior.

The IEGs analyzed here are from two classes. Homer1a and Arc, which have direct effects on neurophysiology, are classified as effectors. Homer1a trafficks and regulates type I metabotropic glutamate receptors [22-25]. Arc trafficks AMPA receptors in an activity-dependent manner [26-29]. The transcription factors Zif268 and c-Fos exert their effects on neurophysiology via regulation of the transcription of downstream genes and, as with Homer1a and Arc, have been shown to be necessary for neuronal plasticity and, in some cases, the consolidation of long-term memory [42-45]. NGFI-B has both direct, proapoptotic effects and transcriptional, mitogenic effects, although its role in neuronal plasticity has not yet been directly assessed [46,47]. As these proteins are regulated by activity and play important roles in synaptic plasticity, their induction illustrates the possible neuroanatomical substrates of conditioned response and the potential for new learning mediated by these circuits (e.g. extinction).

### Palatable food cues elicit locomotor arousal and corticosterone release

Exposure to food-associated cues elicited locomotor hyperactivity, an increase in plasma corticosterone, and enhanced forebrain gene expression. Conditioned locomotor arousal to contexts associated with many drugs of misuse has been documented and is often accompanied by prior sensitization of, or increases in locomotor activity during training [48]. We observed conditioned locomotor arousal in response to contextual cues associated with food in the absence of locomotor sensitization (Figure 2B,C). It is important to determine whether the changes in gene expression that we observe reflect the locomotor component of this conditioned response. Locomotor activity was less often correlated with regional gene expression in the Ensure cues group compared with the water cues group. This lack of correlation cannot be a result of gene expression reaching an upper limit as we have documented higher levels of regional immediate early gene expression in rats with comparable levels of locomotor activity [49]. We have also demonstrated that conditioned locomotor arousal is not sufficient to elicit the pattern of gene expression observed here and that increased gene expression, for example in the prefrontal cortex, is not necessary for locomotor arousal [49-51]. These considerations indicate that the observed patterns of gene expression are not simply caused by increased locomotion.

Exposure to Ensure cues also elicited an increase in plasma corticosterone, possibly due to surprise or frustration elicited in the animals by the expectation of food in a hungry state. The increases in plasma corticosterone reported here are higher than in our previous studies, but are in line with the food-deprivation enhancement of corticosterone release documented in the literature [52]. Although not addressed here, a time course of plasma corticosterone levels following cue exposure would give important information concerning the directionality of the corticosterone response and the activity of the hypothalamo-pituitary-adrenal (HPA) axis in these experiments. The increased number of negative correlations between plasma corticosterone and regional IEG expression in the Ensure cues group suggests that the gene-transcription response may be dampened by circulating corticosterone or another stress-related neuromodulator. Alternatively, forebrain activity on the test day may be involved in suppressing the conditioned activation of the HPA axis. The decreased number of regional gene-expression correlations with locomotion and the increased number of these correlations with plasma corticosterone suggests that neuronal activity in the Ensure cues group is increasingly involved in psychological processes such as controlling stress or anxiety rather than simply a result of increased locomotor activity *per se*. Interestingly, conditioned increases in plasma corticosterone associated with locomotor hyperactivity have also been observed in rats exposed to a cocaine-associated [53] but not a nicotine-associated context [54].

### Food cues alter functional connectivity as assessed by interregional gene expression correlations

Exposure to food-associated contextual cues has been shown to increase locomotor activity and to potentiate food intake by rats, and both of these effects are dependent on an intact prefrontal cortex [55-57]. When rats are allowed to eat beyond satiety in the presence of a discrete cue that had been previously paired with food delivery when hungry, lateral hypothalamus projection neurons in the basolateral amygdala and prefrontal cortex exhibit increased *homer1a *and *arc *expression [58]. Furthermore, basolateral amygdala and prelimbic cortical inputs to the lateral hypothalamus, but not those from the central amygdala or nucleus accumbens, have been demonstrated to be necessary for cue-induced overeating in satiated rats [58-63]. Our gene expression results are consistent with these findings despite some methodological differences. A similar pattern of c-Fos induction to that which we observed was also evident in a recent study assessing regional gene expression in response to shifts in the incentive value of sucrose [64]. These findings suggest that the gene expression changes observed in the present study may play a role in altering the valuation of reward-paired cues used to guide behavior.

**Figure 8:**
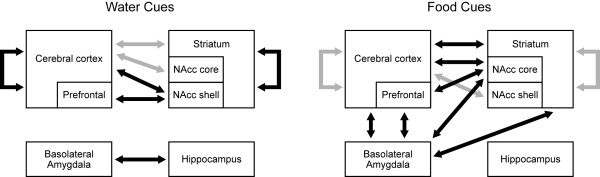
**Circuit diagrams of the regions exhibiting interregional gene-expression correlations elicited by food cues (right) or water cues (left)**. Grey arrows represent relatively fewer numbers of interregional gene-expression correlations and black arrows represent relatively greater numbers of these correlations. Intracortical and intrastriatal interregional gene-expression correlations predominated in the water cues group. In this group, there were relatively few corticostriatal correlations, and cortical engagement of the shell of the nucleus accumbens was greater than that of the core subregion. The water cues group also exhibited correlated gene expression between the hippocampus and the basolateral amygdala. In contrast, the number of intracortical and intrastriatal correlations was relatively smaller and the number of corticostriatal correlations was relatively higher in the food cues group. There was also a shift of cortical correlations to the core of the nucleus accumbens in rats exposed to food cues. Another striking difference between the groups included the loss of the correlated gene expression between the hippocampus and the basolateral amygdala. The basolateral amygdala in the food cues group exhibited a great degree of correlated immediate early gene expression with the cortex and the striatum. These observations suggest that shifts in correlated activity between the cortex and striatum, particularly the nucleus accumbens core, and the basolateral amygdala are involved in the generation of a motivated state by salient external cues. For abbreviations, see legend to Figure 1.

The contrasting patterns of gene expression correlations between the experimental groups suggests that exposure to a food-predictive versus a neutral context selectively recruits and activates specific corticolimbic circuits, resulting in the generation versus suppression of a motivational state. This notion is schematically illustrated in Figure 8. Food-associated cues elicited an increased number of interregional gene expression correlations between the cortex and the basolateral amygdala and the striatum. Of special interest are the correlations between orbitofrontal cortical regions and the basolateral amygdala as these regions cooperate to encode the representations of outcome expectancies based on experience that are used to guide adaptive behavior [65-68]. The coordinated expression of plasticity-related genes in these areas suggests that unreinforced presentation of the conditioned stimuli may prompt an adaptive reevaluation of cued, expected outcomes. Furthermore, the basolateral amygdala may be involved in updating not only affective information but wide-ranging types of encoded feature representations concerning the environment since sensory as well as frontal cortical areas exhibited correlations with this area (but see [69]).

In contrast, gene expression in the basolateral amygdala in the water cues group exhibited no correlations with the cortex but instead exhibited this type of relationship with the dorsal hippocampus (Figure 6C). The hippocampus normally inhibits contextually conditioned locomotor arousal in response to food-paired cues [70]. In addition, varied lines of research have suggested that correlated activity between the hippocampus and basolateral amygdala is involved in learning to suppress conditioned responses and to ignore unimportant events or stimuli [71-75]. As low threshold stimulation of the hippocampus elicits inhibition and higher threshold stimulation elicits excitation of basolateral amygdala projection neurons [76-78], our observations suggest that, in the water-paired context, low levels of hippocampal activity inhibit cortical engagement of the basolateral amygdala while, in the Ensure-paired context, higher levels of hippocampal activity overcomes this feed-forward inhibition, allowing cortical excitation of amygdala output neurons. This type of competition between the cortex and the hippocampus for engagement of the basolateral amygdala has also been proposed to account for the the ability of the cortex to support contextual fear conditioning after pre-training, but not post-training hippocampal lesions [79]. In addition, hippocampus and basolateral amygdala interactions have been implicated in appetitively-driven contextual conditioning [80].

Within the ventral striatum, correlations between the cortex and nucleus accumbens core predominated in the Ensure cues group, as opposed to the nucleus accumbens shell in the water cues group. The nucleus accumbens core, but not the shell, is essential to cue-elicited, goal-directed behaviors including cued reinstatement of cocaine-seeking [81-84]. In contrast, inactivation of the nucleus accumbens shell, but not the core, during acquisition of a rule-based strategy facilitates later set-shifting to another rule-based strategy [81] and also decreases the ability of pre-exposure to inhibit future conditioning to a stimulus known as latent inhibition [85-87]. As the water cues did not have motivational value, the correlations between the cortex and the nucleus accumbens shell in the water cues group may reflect a conditioned inhibitory process or activity within a circuit that mediates the expression of learned irrelevance. These findings suggest that cortical engagement of the striatum and a shift from cortical engagement of the nucleus accumbens shell to the core is associated with conditioned motor arousal indicative of a cue-elicited motivational state.

Although *zif268*, *ngfi-b *and *c-fos *were increased in the diencephalon in the Ensure cues group, there was a paucity of thalamic and hypothalamic interregional gene-expression correlations (Figure 6C). The lack of correlations may simply reflect a decrease in the statistical power of the analyses, as there were fewer usable observations within these structures. With regard to the thalamic gene expression in response to food cues, it appears that the midline and intralaminar nuclei in particular were strongly activated, although critical sensory areas of the thalamus were also involved. As these latter regions transmit primarily taste, visceral, and somatosensory information to the neocortex, one can speculate that this network may be involved in conveying sensory information to the cortical regions that were also strongly activated. In particular, the barrel fields are clearly activated by food cues, suggesting recruitment of sensory, and attentional and executive thalamocortical networks.

Gene expression patterns in the food cues group also indicated an activation of hypothalamic networks including the arcuate, dorsomedial, ventromedial, and lateral hypothalamic regions. This last observation is supported by electrophysiological recordings in non-human primates, showing clear activation of lateral hypothalamic neurons during presentation of food cues only when the animals were hungry [88,89]. In a related study, orexin/hypocretin-positive neurons of the lateral hypothalamus were clearly activated during the expression of food-conditioned or drug-conditioned place preference [90]. Additionally, to our knowledge, this is the first report of cue-elicited activation of the arcuate nucleus of the hypothalamus, where direct energy-balance signals from the periphery (e.g. leptin and ghrelin) are integrated with motor and autonomic effector systems [3,91]. The direct projections from forebrain regions such as the amygdala and cortex to the arcuate nucleus may underlie the cue-elicited activation of this region [92].

### Food cues elicit striatal hnPENK expression

The present study revealed a marked increase in PENK expression in striatal subregions. The striatum receives cortical and thalamic glutamatergic inputs and its gamma-aminobutyric acid (GABA)ergic outputs target motor action systems and premotor brainstem areas, positioning it to organize goal-directed behavior. It is composed primarily of two distinct neuronal populations distinguished by their expressed dopamine receptor subtype and neuropeptide complement and their anatomic projections. One subpopulation expresses the D1 dopamine receptor, dynorphin, and substance P, and projects to the internal segment of the globus pallidus (the "direct pathway") while another expresses the D2 dopamine receptor and enkephalin and projects to the external segment of the globus pallidus (the "indirect pathway"). Cortical or thalamic deafferentation selectively decreases striatal PENK mRNA expression [40,41]. Furthermore, cortical or thalamic excitation results in increases in PENK expression [93]. In addition to the increase in PENK mRNA, excitation of cortical afferents results in immediate early gene expression almost exclusively within enkephalin-containing projection neurons and non-projection interneurons of the striatum [94-101]. Food cues increased corticostriatal IEG correlations and corticothalamic IEG correlations with striatal *hnPENK*, suggesting that food cue-induced activity within cortical and thalamic afferents to the striatum activated the indirect pathway and PENK transcription [102]. This profile is in agreement with our recent results showing specific engagement of the indirect pathway by food-related motivational states [103].

Enkephalin and its μ-receptor have been widely implicated in the hedonic aspects of food and drug intake [104-108]. Stimulation of μ-opioid receptors in the ventral striatum increases the intake of palatable food [36-38,109]. In addition, morphine-paired cues have been shown to increase, while saline-paired cues decrease, extracellular enkephalin levels within the striatum in morphine-conditioned rats [110]. Furthermore, blockade of μ-opioid receptors inhibits cue-elicited alcohol-seeking, methamphetamine-seeking, and food-seeking behaviors [30,32-34]. As striatal enkephalin levels are also highly correlated with recovery of motor function after 6-hydroxydopamine lesions [111-115], these lines of evidence suggest that striatal enkephalin may play a role in the conditioned locomotor arousal to food-associated cues observed in this study. These findings and evidence from recent genetic studies suggests that the corticothalamic activation of the indirect pathway and the resulting enkephalin signaling are intimately linked to conditioned motivated behavior [116,117].

## Conclusion

This is the first report documenting a widespread neuroanatomic activation throughout the diencephalon and telencephalon in response to food-associated cues. Using a novel application of statistical methods, we found that cues associated with palatable food produced dramatic changes in the functional connectivity of circuits that are known to modulate adaptive behavior. Salient (food) cues elicited widespread cortical engagement of the basolateral amygdala, whereas irrelevant (water) cues elicited hippocampal engagement of the basolateral amygdala. In addition, compared with innocuous cues, salient cues increased cortical engagement of the striatum and shifted cortical functional connectivity away from the shell towards the core of the nucleus accumbens. Food cues also increased the striatal expression of enkephalin, a neuropeptide modulator of affective processing. These findings suggest that food-associated cues are capable of powerfully influencing neuronal activity and gene expression in areas mediating higher-order, executive functions and in areas mediating basal functions, such as energy-balance sensing and motor arousal. These processes are integrated to produce behavior, but our understanding of these interactions is incomplete, at best. This work increases our understanding of the powerful effects of food cue-exposure on activity and plasticity-related gene expression within, and functional connectivity between forebrain circuits mediating emotion, cognition, arousal, and behavior.

## Methods

### Subjects and behavioral paradigm

Male Sprague-Dawley rats (*n *= 12 total) used in this study weighed 275–299 g at the beginning of experiment and were housed in pairs in clear plastic cages in an animal colony. Rat chow and water were available in the home cages over the course of the experiment except for a 12–13.5-hour period prior to the final test, during which chow was not available. Lighting in the animal colony was on a 12-h light/dark cycle with lights on 07.00 to 19.00 h. Rats were handled daily for 3 days prior to the beginning of training. All animal care was in strict accordance with institutional animal care and use committee guidelines.

Each rat received access to chocolate Ensure in one of two environments with distinct sensory cues (context A or B) and water in the alternative environment for 15 days (Figure 1A). Contexts A and B differed in olfactory, somatosensory, and visual cues and have been described previously [54]. Training commenced daily in context A between 12.15 and 13.53 hours. Locomotor activity in this context was measured over the 15-day training phase by a photobeam activity system (San Diego Instruments, San Diego, CA, USA). Rats remained in context A for 90 minutes and context B for 30 or 90 minutes daily. Rats remained in context B for 30 minutes on days 3, 10, and 14, and for 90 minutes on all other training days. The restrained time exposure to context B was used to counteract a noted tendency for rats to consume more Ensure in context B. This unexplained tendency could be a result of different motivational states generated by the different training environments [118]. Alternatively, this tendency could be explained by a diurnal difference in motivational state, as exposure to context B during training always occurred later in the day. Regardless of the reason, decreasing the amount of time that the rats had access to Ensure in this environment therefore equalized the absolute amount of Ensure that both of the experimental groups consumed during the training period. This manipulation, however, introduced another variable to be considered on the test day. We cannot completely rule out an effect of an incomplete phase shift caused by the limited access to Ensure in context B on training days 3, 5, and 14 on test day locomotor activity, plasma corticosterone, or gene expression. We find this to be a very unlikely interpretation of the data as these limited exposures occurred on only 3 of the 15 training days. In addition, within these limited exposures, rats in context B consumed almost two-thirds of the Ensure that rats in context A consumed. We feel that these considerations make it unlikely that the effects observed on the test day are due to differences in phase shifts between the groups.

Three days after training, all rats were re-exposed to context A, where half of the rats had previously had access to Ensure and the other half had access to water. No bottles were present on the test day. Stressors can paradoxically increase appetitite-based conditioned responding [20,34,49,119-122]. We, therefore, used acute food-deprivation as a means of enhancing food cue-induced, appetitite-motivated behavior and neuronal activity by removing rat chow from home cages 12–13.5 hours prior to testing in context A [4,123]. On the test day, after 45 minutes in context A, where locomotor activity was again measured (in five 9-minute bins), rats were anesthetized with halothane and rapidly decapitated for tissue collection. Exposure of pairs of rats from the water cues and Ensure cues groups to context A on the test day was conducted from 12.15 to 14.30 hours, a period of time that overlapped with the time spent in both contexts A and B during training. Therefore, any effects observed cannot simply be accounted for by a diurnally entrained rhythm established during training.

### Tissue isolation, enzyme-linked immunoassay, and ISH procedures

The procedures utilized for tissue isolation, corticosterone enzyme-linked immunoassay, and ISH have all been described previously [54]. The corticosterone result from one of the rats in the water cues group was not included in the statistical analysis because it was derived from a hemolyzed sample and was determined to be an outlier via a Grubbs outlier test (G_1 _= 1.858 > 1.822 at 95% C.I.). Intra-assay variability was <10%.

Templates for riboprobes were amplified from a rat-brain cDNA library. The primers used for the generation of antisense probe templates for all of the genes analyzed, except for c-*fos*, are listed in Additional file 5. Designing a probe against intron 1 of the *PENK *gene allowed us to measure *hnPENK *levels, which are temporally more proximal to and more sensitive to relative changes in *PENK *transcription, as we avoided detecting the large cellular pool of *PENK *mRNA that exists in striatal, indirect pathway neurons. A BLAST search was conducted with each of the predicted template sequences in order to insure that probes did not detect other known mRNA species. Appropriately sized bands were excised from 1.2% agarose after electrophoresis and purified with a gel extraction kit following the manufacturer's protocol (Qiagen, Valencia, CA, USA). *In vitro *transcription reactions using either amplified templates or a linearized plasmid containing c-*fos *sequence (kindly provided by T. Curran) were used to generate ^35^S-labeled antisense probes and the ISH procedure were carried out as described previously [54].

Sections run through the ISH procedure were exposed to a phosphorimager screen for between 4 and 7 days. The exposed screens were read in a Typhoon scanner and quantification of signal in particular brain regions (Figure 1B) was performed using ImageQuant software (both Molecular Dynamics Sunnyvale, CA, USA). Values for each hemisphere were averaged in order to arrive at a single value for each region and each rat. The values for the anterior and posterior cingulate cortex and the CA1 and CA3 fields of the dorsal hippocampus were averaged to arrive at single values for these regions. Background correction was determined to be unnecessary, as all tissue for each gene was run through the ISH procedure, including phosphor-screen exposure and scanning, and there were no observed differences in white matter signal intensities between groups. All measurements fell well within the ~10,000-fold linear range of the phosphor screen and Typhoon scanner used here, as determined by running a set of standards with known amounts of radioactivity in triplicate on the phosphor screen and running a correlation analysis and Z-test. After phosphor-screen exposure, slides were counterstained with Nissl stain and dehydrated through a graded series of ethanol and xylene. A coverslip was then applied. Images were taken with a Leica DC 300F digital camera linked to Image Pro-Plus software on a PC through a Leica DMRX microscope. Using Photoshop software, these images were scaled and superimposed upon phosphorescence images from which pixel intensity measurements were taken for anatomical confirmation of regional measurements. Examples of regions of phosphorimages from which measurements were taken with photomicrographs of paired, Nissl-stained sections can be found in Additional files 4, 5, and 6.

Although a time course of gene expression was not completed in this study, multiple experiments have demonstrated that peak induction of these early response gene transcripts occurs around 30 minutes after behavioral and pharmacological stimulation [124-126], thus we have probably detected mRNA levels resulting from levels of transcription peaking around 15 minutes of exposure to context A. Neither of the groups exhibited significant differences in locomotor activity during this period (Figure 2C), again suggesting that the transcriptional response is not purely due to differences in locomotion.

### Statistical analyses

Behavioral data from the activity cages were analyzed using StatView software (SAS Institute, Cary, NC, USA). Total horizontal activity measurements in context A over the 15-day training period and on the test day were analyzed with a two-factor, between-group and within-group analysis of variance (ANOVAs) with treatment as the between-subjects factor and days or time interval as the within-subjects factor. Similar tests were run for daily Ensure and water intake and daily weights over the course of the experiment. After determining that a significant treatment effect existed for the test day locomotor activity data, comparisons between individual groups within each 9-minute time interval were performed using a one-way, between-subjects ANOVA. Test day plasma corticosterone data was analyzed with a one-factor ANOVA with treatment as the between-subjects factor.

For the ISH data, the averaged pixel intensities for each region were entered into StatView and were analyzed by first performing a two-way between and within subjects ANOVA with cues as the between-subjects factor and brain region as the within-subjects factor. After determining whether a significant group effect was present, comparisons between individual regions were performed using a one-way, between-subjects ANOVA.

Recent genetic evidence suggests that the expression of individual IEGs is under the control of overlapping but unique complements of transcription modulating entities [127]. Additionally, as each transcript gave a unique expression pattern, we used each IEG expression measurement as an individual observation, yielding greater statistical power for correlation analyses. In this light, we conducted correlation analyses of regional gene expression with total horizontal activity, plasma corticosterone, and gene expression within other regions. These correlations were conducted in order to ascertain whether alterations in locomotor acitivity and corticosterone levels were related to regional changes in activity-dependent gene expression, and to assess whether exposure to the Ensure-associated context was associated with changes in correlated neuroanatomical activity-dependent gene expression (i.e. functional connectivity).

We began by normalizing each regional gene expression measurement in the Ensure cues and water cues groups to the mean water cues group expression value. For example, imagine an experiment where n = 3 per group, and rats exposed to water cues had 1, 2, and 3 (mean = 2) arbitrary units of gene expression in region *x*, and rats exposed to Ensure cues had 2, 3, and 4 (mean = 3) arbitratry units of gene expression in the same region. The average control value of 2 would be used to divide all individual measurements for that gene in region *x *in both groups. The rats exposed to water cues would then have 0.5, 1.0, and 1.5 (new mean = 1.0) units and the rats exposed to Ensure cues 1.0, 1.5, and 2.0 (new mean = 1.5) units of gene expression within this region. This process allowed us to plot the measurements of different genes within a single region on a common axis. The regional, normalized *homer1a*, *arc*, *zif26*8, *ngfi-b*, and *c-fos *expression measurements were used to calculate Pearson product moment correlations with locomotor activity, plasma corticosterone, and gene expression in other regions within the water and Ensure cues groups. Correlation coefficients were computed within each group, using only the genes within a region that exhibited a mean difference between the Ensure cues and water cues groups as revealed by the ANOVA means analysis. We excluded regional measurements of gene expression that exhibited no difference between groups, because the upstream signaling processes responsible of the transcription of that gene within that region cannot be involved in the generation of the differential motivational state. This procedure decreased the probability the occurrence of type II errors. Correlation Z-tests were applied to all the correlations in order to determine the significance of any relationships. To decrease the probability of the occurrence of type I errors resulting from outliers, we subjected each interregional gene expression correlation to a jackknife procedure in which each measurement was removed and correlations were recomputed without that single observation. Correlations were considered to be reliably significant if they remained significant (*p *< 0.05) throughout every iteration of this jackknife procedure. This procedure is similar to the technique used to map functionally activated circuits with fluorodeoxyglucose [128,129]. Correlations were also computed between regional extrastriatal IEG expression and striatal *hnPENK *expression within each group. This analysis was meant to explore activity within afferent regions that may contribute to the transsynaptic induction of striatal *hnPENK *expression.

## Authors' contributions

CAS contributed to the conception and design of the project, analysis and interpretation of the data, carried out the studies, and drafted the manuscript. QZB contributed to the design of the project, and interpretation of the data, and conducted portions of the studies. CFL contributed to the conception and design of the project, interpretation of the data, and the draft of the manuscript. AEK contributed to the conception and design of the project, the interpretation of the data, and the draft of the manuscript.

## Supplementary Material

Additional File 1 **Supplementary Table 1**. Detailed statistics of regional ANOVAs for each gene and area analyzed. Areas in bold were significantly different between the water cues and Ensure cues groups. For abbreviations, see legend to Figure 1. Click here for file

Additional File 2 **Supplementary Figure 1**. Telencephalic expression of *arc *induced by exposure to water or Ensure cues. (**A**) Pseudocolor autoradiographic phosphorescence images of coronal brain sections hybridized with a probe *for arc *from a rat in the water cues group (top) and the Ensure cues group (bottom). (**B**) Graphical representation of semiquantitative measurements of *in situ *hybridization *for arc *in telencephalic regions. Ensure cues increased the expression *of arc *in many of the corticolimbic regions examined (*p < 0.05). For abbreviations, see legend to Figure 1. Click here for file

Additional File 3 **Supplementary Figure 2**. Telencephalic expression of *ngfi-b *induced by exposure to water or Ensure cues. (**A**) Pseudocolor autoradiographic phosphorescence images of coronal brain sections hybridized with a probe for *ngfi-b *from a rat in the water cues group (top) and the Ensure cues group (bottom). (**B**) Graphical representation of semiquantitative measurements of *in situ *hybridization for *ngfi-b *in telencephalic regions. Ensure cues increased the expression of *ngfi-b *in a many of the corticolimbic regions examined (*p < 0.05). For abbreviations, see legend to Figure 1. Click here for file

Additional File 4 **Supplementary Figure 3**. Telencephalic expression of the transcription factor c-*fos *induced by exposure to Ensure cues. (**A**) Pseudocolor autoradiographic phosphorescence images of coronal brain sections hybridized with a probe for c-*fos *from a rat in the water cues group (top) and the Ensure cues group (bottom). (**B**) Graphical representation of semiquantitative measurements of *in situ *hybridization for c-*fos *in telencephalic regions. Ensure cues increased the expression of c-*fos *in a many of the corticolimbic regions examined (*p < 0.05). For abbreviations, see legend to Figure 1. Click here for file

Additional File 5 **Suplementary Table 2**. Primer sequences used to generate template cDNA for the generation of riboprobes. A T7 recognition sequence (5'-CAGAGATGCATAATACGACTCACTATAGGGAGA-3') was added to the 5' end of each reverse primer for use in generating the radiolabeled antisense probe. Numbers in parentheses after the primer sequence represent the base number as defined by the UniGene database sequence denoted for each gene. Click here for file

Additional File 6 **Supplementary Figure 4**. Phosphorimages from which nucleus accumbens *homer1a *expression measurements were taken with their Nissl-stained counterparts for verification of anatomical placements. (**A**) and (**B**) are from a rat exposed to water cues. (**C**) and (**D**) are from a rat exposed to Ensure cues. ac, anterior commisure; LV, lateral ventricle; NAcc, nucleus accumbens. Click here for file

Additional File 7 **Supplementary Figure 5**. Phosphorimages from which amygdala *arc *expression measurements were taken with their Nissl-stained counterparts for verification of anatomical placements. (**A**) and (**B**) are from a rat exposed to water cues. (**C**) and (**D**) are from a rat exposed to Ensure cues. BLA, basolateral amygdala; CeA, central nucleus of the amygdala. Click here for file

Additional File 8 **Supplementary Figure 6**. Phosphorimages from which hypothalamic *zif268 *expression measurements were taken with their Nissl-stained counterparts for verification of anatomical placements. (**A**) and (**B**) are from a rat exposed to water cues. (**C**) and (**D**) are from a rat exposed to Ensure cues. DMH, dorsomedial hypothalamus; VMH, ventromedial hypothalamus. Click here for file
