# Supplementary figures and images for: Food-associated cues alter forebrain functional connectivity as assessed with immediate early gene and proenkephalin expression
Source: BMC Biol. 2007 Apr 26;5:16. doi: 10.1186/1741-7007-5-16 (PMC1868707; doi:10.1186/1741-7007-5-16)

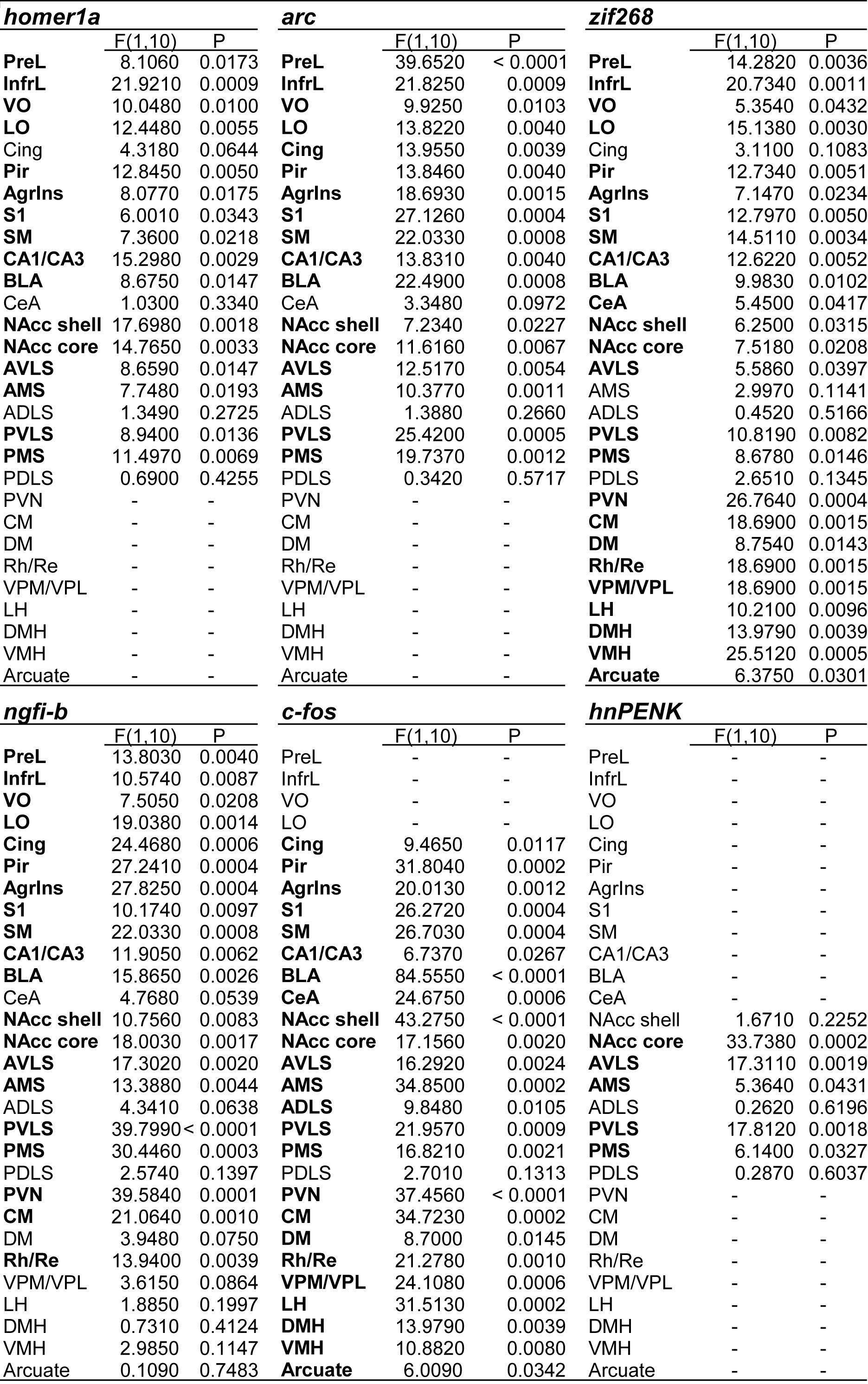

Supplement: Additional File 1 — Supplementary Table 1. Detailed statistics of regional ANOVAs for each gene and area analyzed. Areas in bold were significantly different between the water cues and Ensure cues groups. For abbreviations, see legend to Figure 1. [file 1741-7007-5-16-S1.tiff]

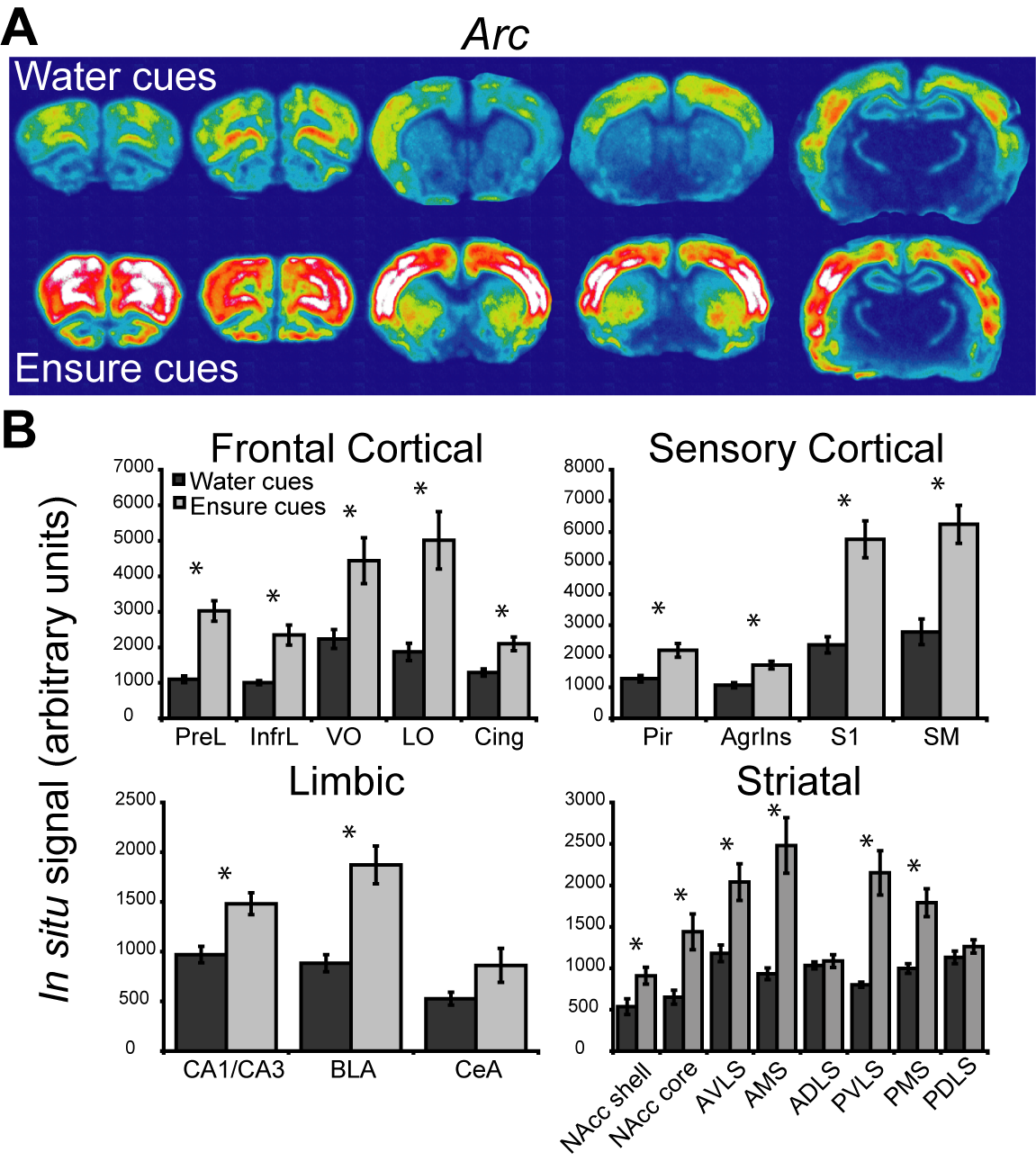

Supplement: Additional File 2 — Supplementary Figure 1. Telencephalic expression of arc induced by exposure to water or Ensure cues. (A) Pseudocolor autoradiographic phosphorescence images of coronal brain sections hybridized with a probe for arc from a rat in the water cues group (top) and the Ensure cues group (bottom). (B) Graphical representation of semiquantitative measurements of in situ hybridization for arc in telencephalic regions. Ensure cues increased the expression of arc in many of the corticolimbic regions examined (*p < 0.05). For abbreviations, see legend to Figure 1. [file 1741-7007-5-16-S2.tiff]

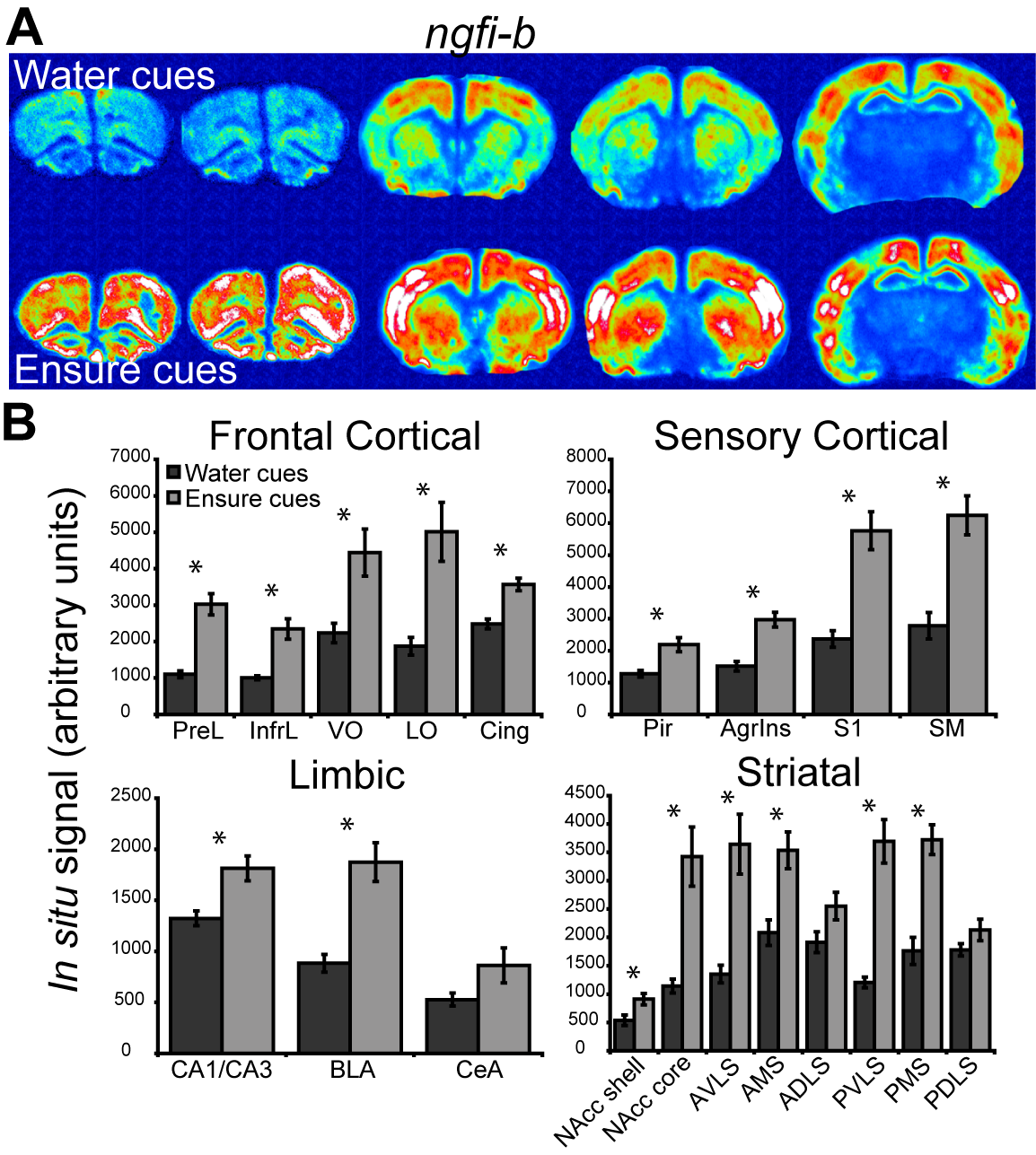

Supplement: Additional File 3 — Supplementary Figure 2. Telencephalic expression of ngfi-b induced by exposure to water or Ensure cues. (A) Pseudocolor autoradiographic phosphorescence images of coronal brain sections hybridized with a probe for ngfi-b from a rat in the water cues group (top) and the Ensure cues group (bottom). (B) Graphical representation of semiquantitative measurements of in situ hybridization for ngfi-b in telencephalic regions. Ensure cues increased the expression of ngfi-b in a many of the corticolimbic regions examined (*p < 0.05). For abbreviations, see legend to Figure 1. [file 1741-7007-5-16-S3.tiff]

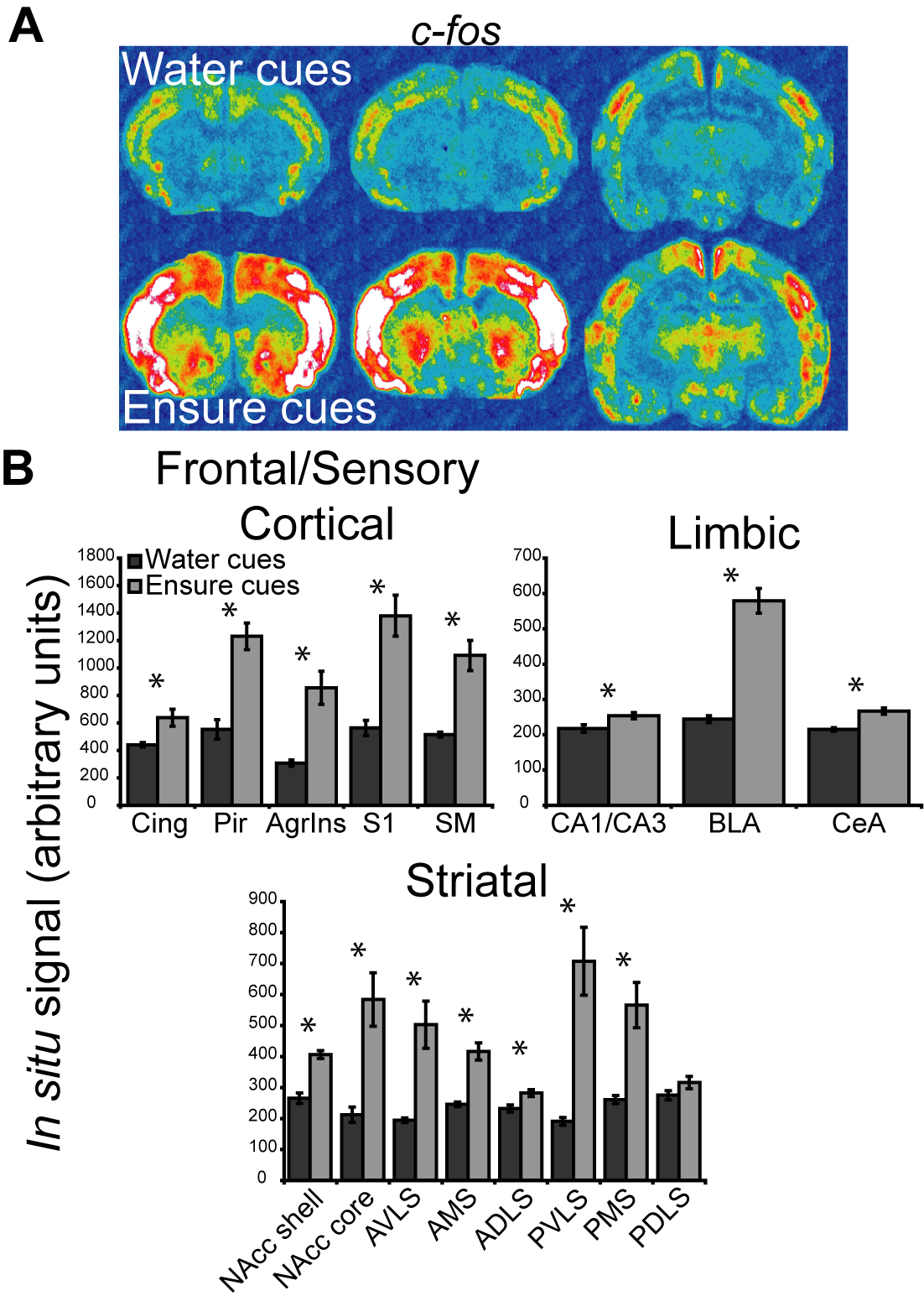

Supplement: Additional File 4 — Supplementary Figure 3. Telencephalic expression of the transcription factor c-fos induced by exposure to Ensure cues. (A) Pseudocolor autoradiographic phosphorescence images of coronal brain sections hybridized with a probe for c-fos from a rat in the water cues group (top) and the Ensure cues group (bottom). (B) Graphical representation of semiquantitative measurements of in situ hybridization for c-fos in telencephalic regions. Ensure cues increased the expression of c-fos in a many of the corticolimbic regions examined (*p < 0.05). For abbreviations, see legend to Figure 1. [file 1741-7007-5-16-S4.tiff]

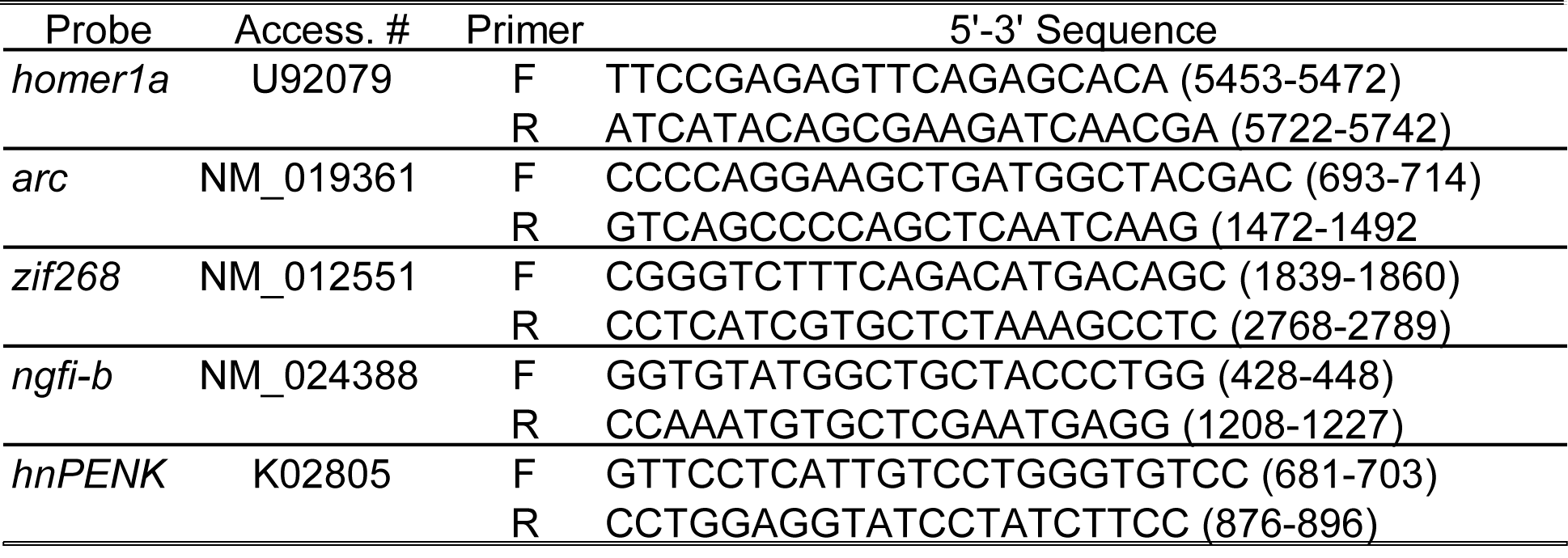

Supplement: Additional File 5 — Suplementary Table 2. Primer sequences used to generate template cDNA for the generation of riboprobes. A T7 recognition sequence (5'-CAGAGATGCATAATACGACTCACTATAGGGAGA-3') was added to the 5' end of each reverse primer for use in generating the radiolabeled antisense probe. Numbers in parentheses after the primer sequence represent the base number as defined by the UniGene database sequence denoted for each gene. [file 1741-7007-5-16-S5.tiff]

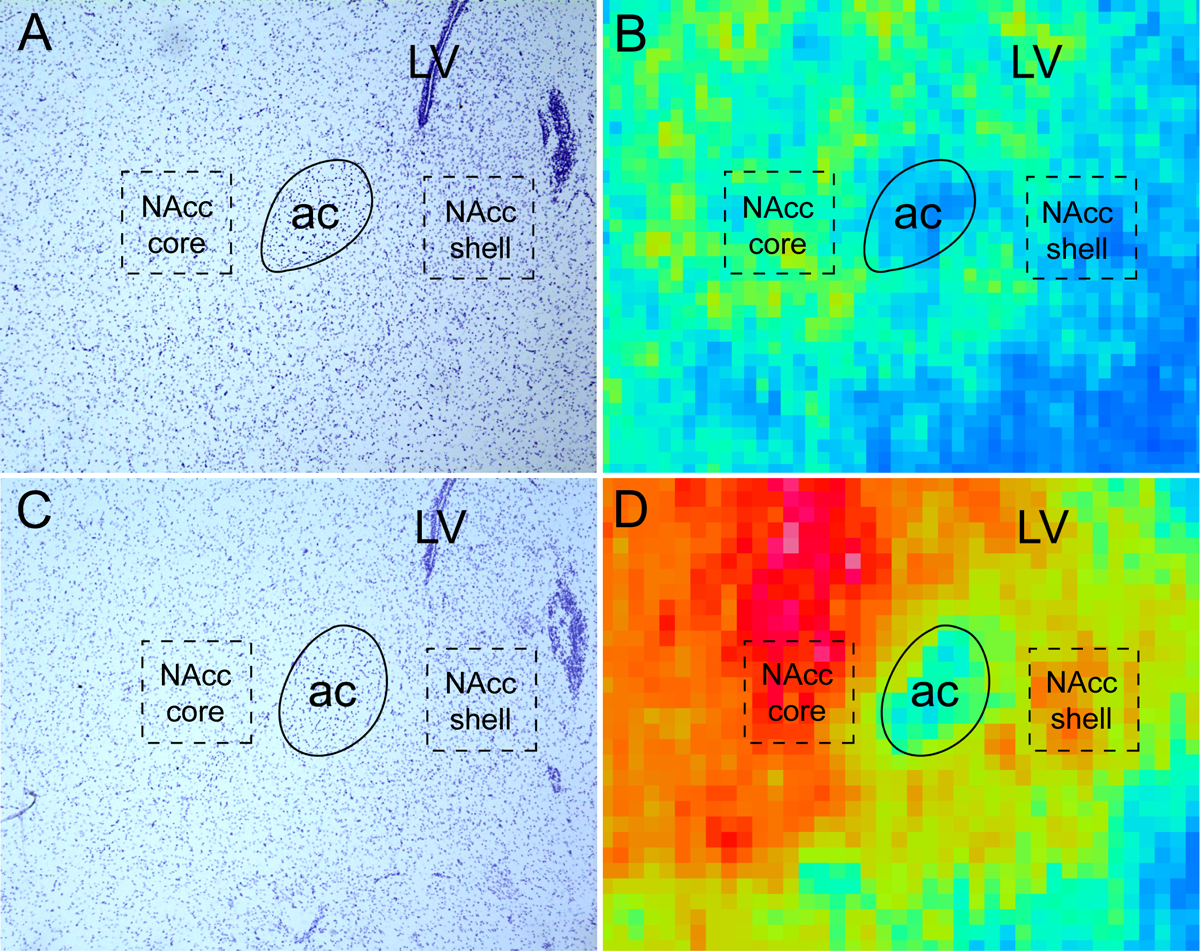

Supplement: Additional File 6 — Supplementary Figure 4. Phosphorimages from which nucleus accumbens homer1a expression measurements were taken with their Nissl-stained counterparts for verification of anatomical placements. (A) and (B) are from a rat exposed to water cues. (C) and (D) are from a rat exposed to Ensure cues. ac, anterior commisure; LV, lateral ventricle; NAcc, nucleus accumbens. [file 1741-7007-5-16-S6.tiff]

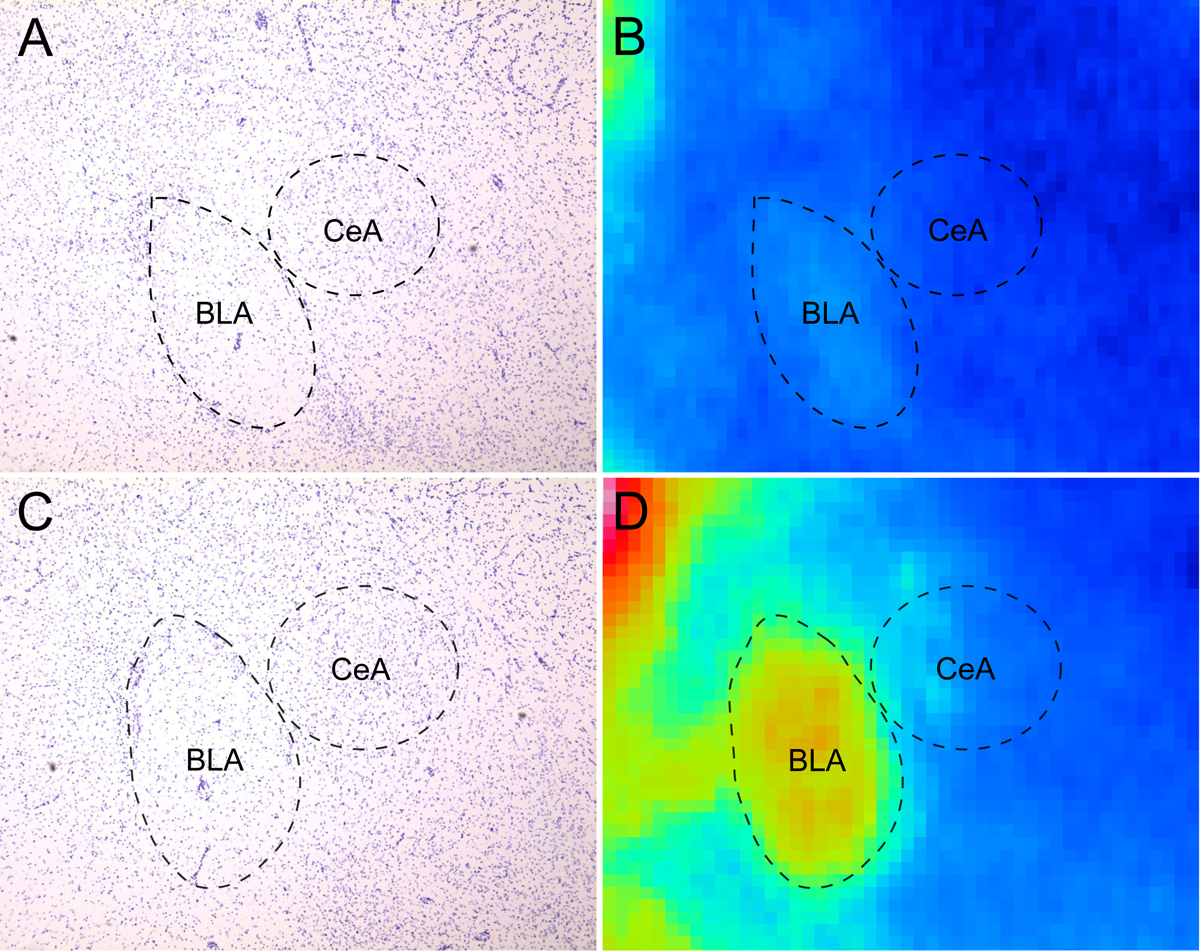

Supplement: Additional File 7 — Supplementary Figure 5. Phosphorimages from which amygdala arc expression measurements were taken with their Nissl-stained counterparts for verification of anatomical placements. (A) and (B) are from a rat exposed to water cues. (C) and (D) are from a rat exposed to Ensure cues. BLA, basolateral amygdala; CeA, central nucleus of the amygdala. [file 1741-7007-5-16-S7.tiff]

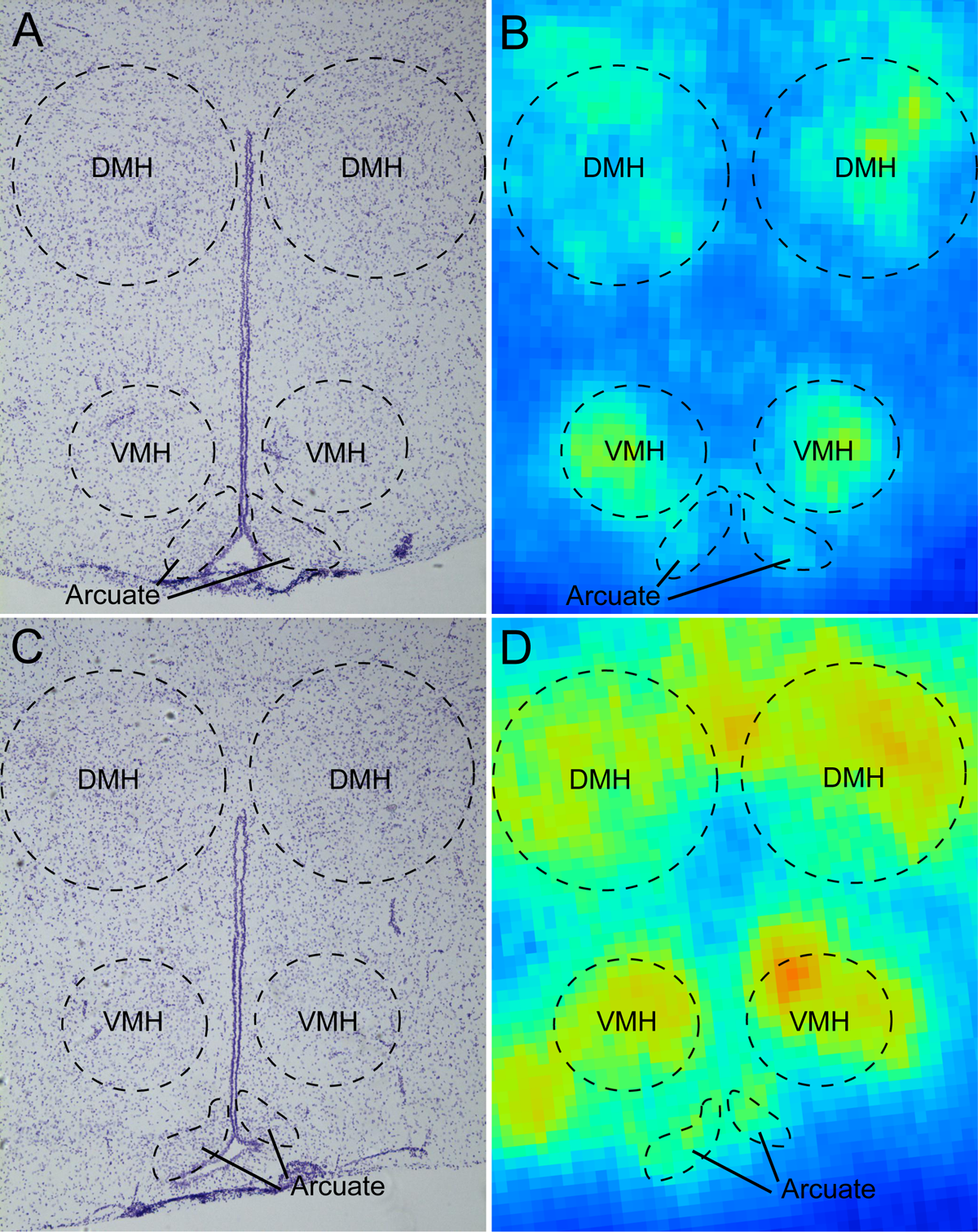

Supplement: Additional File 8 — Supplementary Figure 6. Phosphorimages from which hypothalamic zif268 expression measurements were taken with their Nissl-stained counterparts for verification of anatomical placements. (A) and (B) are from a rat exposed to water cues. (C) and (D) are from a rat exposed to Ensure cues. DMH, dorsomedial hypothalamus; VMH, ventromedial hypothalamus. [file 1741-7007-5-16-S8.tiff]
